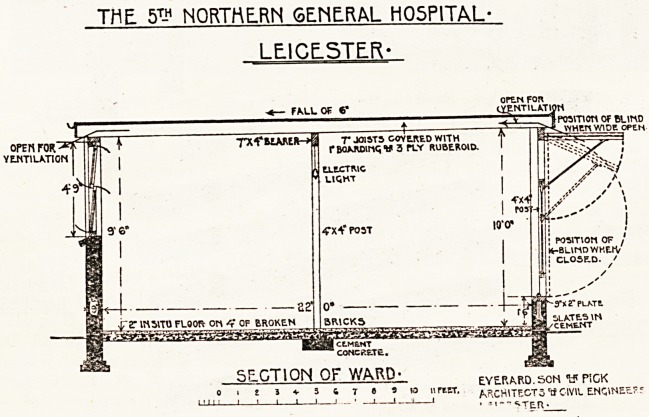# The Fifth Northern General Hospital, Leicester: The Recent Additions

**Published:** 1915-10-23

**Authors:** 


### THE FIFTH NORTHERN GENERAL HOSPITAL, LEICESTER.

### The Recent Additions.

(By THE ARCHITECTS.)

On March 18 a deputation from Leicester visited the War Office and had an interview with Sir Alfred Keogh for the purpose of placing before the Director-General the desirability of extending the base hospital in preference to using a number of schools and other buildings in the town, and at this meeting promises were made to carry out the extension of the buildings in twelve weeks from the date of instructions. These instructions were actually received on Sunday, March 21. In the four following days drawings were prepared, quantities got out, and on Thursday, the 25th, eight tenders for the proposed works were placed before the War Office authorities. The tender submitted by Messrs. Henry Herbert and Sons, of Leicester, amounting to £8,357, was accepted, and the contract was signed on the following day, March 26, and the work commenced. The extension for 530 beds was efficiently carried out in the short time of one day under eight weeks. The terms of the contract allowed ten weeks, consequently a bonus of £10 per day for fifteen days was earned by Messrs. Henry Herbert and Sons, and this amount (£150) the builders generously gave to the workmen who were employed on the job. The new wards are of the open-air type, with provision for fifty-two beds in each large ward and one bed in a separation ward. At the end of the large ward a day space is provided, having a fireplace. This is found useful for convalescent patients and also for dining purposes. The wards are built on a site sloping to the south-east and are placed 30 feet clear apart.

The wards are connected up by a wide covered way which intersects the centre of each double block. It may be mentioned, although these wards are of a semi-permanent character, that the cost (£14 per head) is about the same as if they were built with galvanised iron or asbestos sheeting.

View Showing Blinds.

1 2 3

(1) The End Screen to Keep out Sun and Side Draughts. (2) Blinds Folding Up. (3) Blind Closed for Night Use or very Rough Weather.

In addition to the hospital extensions, extensions of the nurses' home for 136 beds, together with new kitchen arrangements, have also been carried out. The cooking for nurses is now, therefore, entirely separated from that of the patients, and this method has in practice been found to work better than the joint arrangement which was formerly in operation.

Description of Open-air Wards.

The open-air wards are the chief interest in the extension, and have been planned and erected as five double blocks each for 106 beds, making 530 beds in all. They cost complete £1,650 each pair of wards. The wards are constructed with common brick walls; the floors are cement *in situ*; the roofs 7-inch joists about 2 feet apart covered with 1-inch boarding and 3-ply ruberoid. A centre line of posts with a 7-inch by 4-inch beam allows the wood joists to be kept a reasonable size, and the posts are useful to hang up the patients' charts. The windows are casements hung on centres. The fronts of the wards are to the south-east and, excepting the day and dining space at the ends of wards, they are entirely open. We have devised folding canvas shutter blinds which can be opened horizontally at two lower angles, or folded down and entirely closed. The angle at which the blind is required is arranged by a peg on the revolving side-irons, and the blinds are securely fastened when closed by hasps on the posts to each half. By this method the flapping of blinds has been prevented; they fit closely at the sides and bottoms, present a neat appearance, and up to the present time have proved very successful in every way. It will be seen that when the shutters are closed ample cross-ventilation is provided by the open spaces between the roof joists. These open spaces are protected against driving rains by fascia-boards. In addition to this cross-ventilation the canvas also permits air to pass through.

Two-decker Beds.

The additional accommodation has been made for orderlies by putting in the existing blocks two-decker beds. Considering the ample cross-ventilation provided, this arrangement was at the time thought sufficient, but experience of the plan proves that this provision is not quite good enough. The best thing in its favour which can be fairly said is that the men are better provided for than if they were under canvas. We are glad to hear the War Office has condemned two-decker beds.

Other New Features.

The sanitary arrangements were provided on a liberal scale in the first instance, and are answering the purposes of the increased number of orderlies satisfactorily. The only addition to these buildings has been a barber's shop, and this has been erected between the ablution block and the canteen. A second operation theatre has been provided in another detached building as shown on the plan, but these theatres are still somewhat poor accommodation for the very important work which has to be done, and it would be well to provide a new theatre block properly arranged. More motor sheds, a proper pathological block, and other works are under consideration by the War Office authorities at the present time.

## Figures and Tables

**Figure f1:**
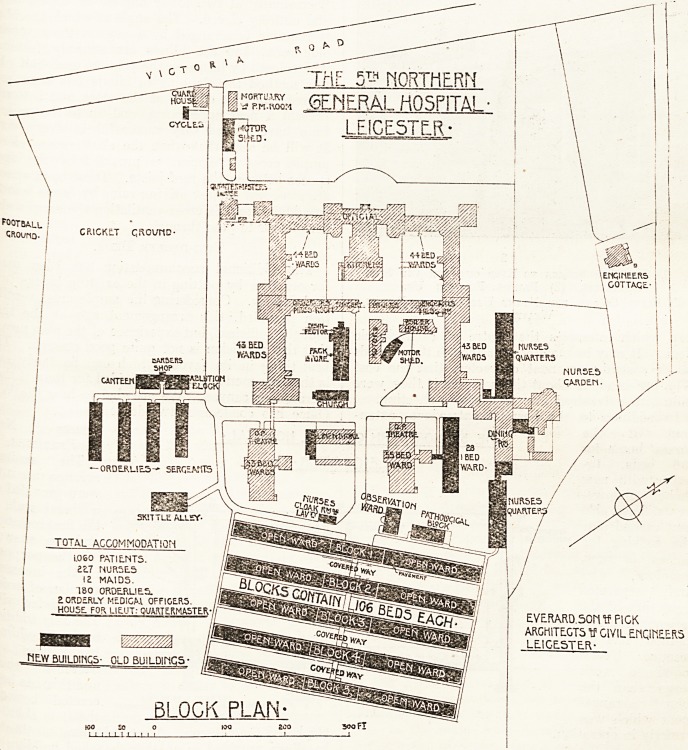


**Figure f2:**
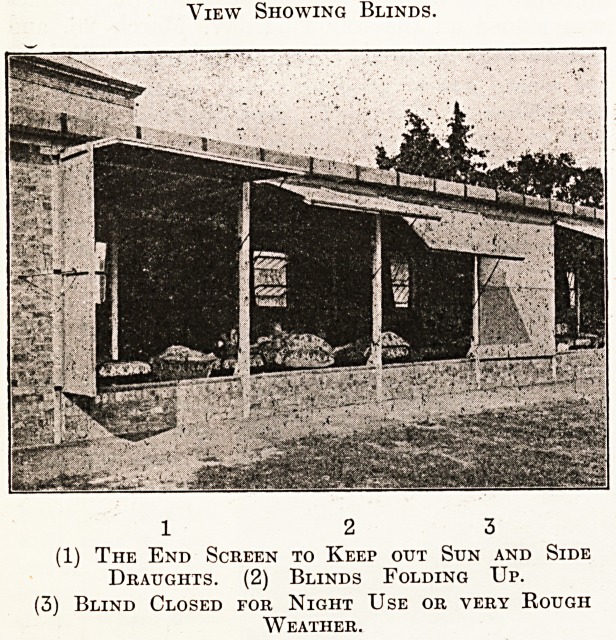


**Figure f3:**